# Antimelanoma Activity of the Mastoparan Peptides MPX and MP1: Cellular Selectivity and Mechanism of Action

**DOI:** 10.1021/acs.molpharmaceut.6c00083

**Published:** 2026-04-24

**Authors:** Amanda Sansone Semerdjian, Manoel Arcisio-Miranda

**Affiliations:** Laboratório de Neurobiologia Estrutural e Funcional (LaNEF), Departamento de Biofísica, Escola Paulista de Medicina, Universidade Federal de São Paulo, São Paulo, SP 04023-060, Brazil

**Keywords:** cationic peptides, mastoparan, melanoma, fluorescence microscopy, selectivity

## Abstract

MPX (Mastoparan X) and MP1 (Polybia-MP1) are cationic amphiphilic peptides isolated from the venom of insects of the *Vespidae* family. Due to their physicochemical features, these peptides show affinity for biomimetic membranes, especially the more anionic ones. In this study, we investigated the cell-selective effects of MPX and MP1 peptides, comparing their cytotoxicity and metabolic effects on melanocytes and their respective counterparts, melanoma cells. By employing UV–vis spectroscopy and fluorescence microscopy assays, we provided mechanistic insights into these peptides’ antimelanoma activity. MPX and MP1 peptides were cytotoxic to both cell lines. However, their effect was more intense in melanoma than in melanocyte cells. Both cell lines treated with MPX and MP1 peptides showed phosphatidylserine externalization. However, unlike MP1 treatment, MPX also elicited pore formation in the plasma membranes of melanoma cells. These data indicate that the mode of action of MPX and MP1 involves different processes, namely, necrosis and apoptosis, respectively. The levels of reactive oxygen species did not undergo significant changes, but intracellular calcium levels increased considerably in melanoma cells treated with MPX. Both cell lines have their mitochondrial membrane potentials decreased after peptide treatments; nonetheless, this reduction was more intense in melanoma than in melanocyte cells. Altogether, these data reveal that the MPX and MP1 peptides are selective for melanoma tumorigenic cells, reassuring their antimelanoma therapeutic potential. Despite their structural similarities, these peptides have cell-type-relatable distinct modes of action, which resonate in both plasma membrane alterations and metabolic processes, indicating that these effects are not mutually exclusive and not the same for all cells and conditions.

## Graphical abstract

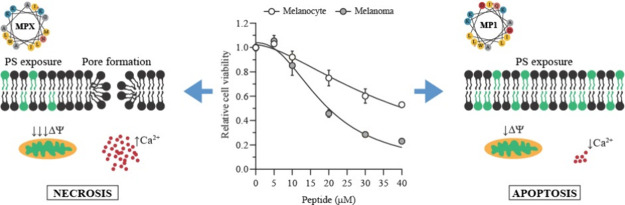

## Introduction

Cationic amphiphilic peptides (CAPs) are amphipathic, positively charged molecules composed of up to 40 amino acid residues structurally organized in α-helical, β-sheet, loop, or extended conformations.^1^ They are found in microorganisms, plants, and animals as defensive effectors of their immune system.^2^ They can have antibacterial, antioxidative, antifungal, and antitumor activities.^3–5^ Therefore, recent studies point out CAPs as potential therapeutic agents.^6,7^

Mastoparan is a class of CAPs originally described for promoting degranulation and histamine release in mast cells.^8,9^ Most mastoparans have 14 amino acid residues, including the peptides discovered in social wasps, MPX (Mastoparan X) and MP1 (Polybia-MP1).^10,11^ Despite having the same length, these peptides differ from each other in terms of hydrophobicity, hydrophobic moment, and net charge.Table 1 shows their primary sequences as well as their physicochemical features. Both peptides share the same number of apolar and basic amino acid residues, which segregate to form an ideal amphipathic structure for interacting with the membrane interface. The α-helical conformation adopted by these peptides when in contact with membranes may be stabilized by their amidated C-terminal, as already demonstrated for other CAPs.^12–14^ Additionally, at physiological pH, MPX exhibits a net electrical charge of +4 and an MP1 of +2.

**Table 1:** Primary Sequence and Physicochemical Properties of Peptides MPX and MP1 at pH 7.4^a^

**peptide**	**sequence (N–C)**	**H**	**μH**	**NC**
MPX	INWKGIAAMAKKLL-NH_2_	0.560	0.419	4
MP1	IDWKKLLDAAKQIL-NH_2_	0.489	0.511	2

a The abbreviations stand for hydrophobicity (H), hydrophobic moment (μH), and net charge (NC). Data was generated using HeliQuest.^15^

Previous studies suggest that in addition to peptide hydrophobicity, peptide net charge plays a crucial role in the peptide-cell membrane electrostatic interaction, especially in the case of membranes exhibiting a high negative surface potential.^16^ Cancer cells present more anionic compounds on their surface than healthy cells, resulting in an overall more negatively charged membrane.^17^ In the domain of biomimetic membranes, it is generally accepted that the anticancer activity of mastoparans arises from their amphipathic and cationic nature, which promotes favorable interactions with cancer cell-like membranes.^7^ However, it has previously been observed with in vitro experimental models that peptides MPX and MP1 can either induce necrosis^16,18^ or apoptosis,^19^ depending on the cell type. Therefore, we propose that peptide anticancer activity investigation must consider not only the peptide structure *per se* but also the complex plasma membrane composition of living cells.

Plasma membranes are fluid and dynamic structures composed of an asymmetric lipid bilayer with embedded proteins and carbohydrate moieties.^20^ This organization may vary according to cell microenvironment.^21,22^ Supporting this notion, studies have reported an association between membrane organization and tumor progression in melanoma cells.^23,24^ Melanoma is the most lethal type of skin cancer.^25^ In 2022, approximately 60,000 people worldwide died due to cutaneous melanoma.^26^ Early-stage melanomas can be surgically removed and cured, but advanced-stage melanomas require systemic treatments that frequently lead to adverse events and toxicities.^27^ Hence, the development of more selective drugs for this disease is important.

In the present study, we have investigated the cytotoxic effects of the peptides MPX and MP1 on melanocyte (Melan-a) and melanoma (B16–F10) murine cells as well as their metabolic outcomes. The melanocyte cells and their respective cancerous counterparts were chosen to enable more reliable comparisons than those of nonrelatable cell types. Thereby, we have demonstrated that both peptides are cell-selective, reducing the viability of melanoma cells more pronouncedly. Additionally, our findings reveal that the peptides’ mode of action may not be the same for all cells and conditions, challenging the common belief that molecules invariably adopt a single mechanism of action. By fluorescence microscopy approaches, we have demonstrated that MPX triggers necrosis, whereas MP1 induces apoptosis in melanoma cells. In melanocytes, both peptides trigger apoptosis. These findings corroborate previous studies, indicating the antitumoral selective action of mastoparans and enlightening their molecular mechanism and cell type-dependent effects.

## Materials and Methods

### Chemicals and Reagents

Sigma-Aldrich Co. (S. Louis, MO, USA) supplied Roswell Park Memorial Institute Medium 1640 (RPMI-1640), 3-(4,5-dimethyl-thiazol-2-yl)-2,5-diphenyltetrazolium bromide (MTT), propidium iodide (PI), phorbol-12-myristate-13-acetate (PMA), and other chemicals used to prepare buffer solutions. Labsynth (Diadema, SP, Brazil) supplied dimethyl sulfoxide (DMSO). Thermo Fisher Scientific (Waltham, Massachusetts, USA) supplied fluorescence probes Annexin V-FITC conjugate (AV-FITC), dihydroethidium (DHE), Fluo-4, Hoechst 33342, tetramethylrhodamine ethyl ester perchlorate (TMRE), fetal bovine serum (FBS), penicillin-streptomycin solution, and UltraPure DNase/RNase-free distilled water. The Hepes-buffered solution used in fluorescence experiments was prepared with ultrapure distilled water (Merck Millipore, MA, USA) and 20 mmol L^–1^ of Hepes, 118 mmol L^–1^ of NaCl, 5 mmol L^–1^ of KCl, 1.1 mmol L^–1^ of MgSO_4_, 1.1 mmol L^–1^ of KH_2_PO_4_, 2.5 mmol L^–1^ of CaCl_2_, and 5.1 mmol L^–1^ of α-d-glucose at pH 7.4. The binding buffer solution used in AV-FITC assays was prepared with ultrapure distilled water and 10 mmol L^–1^ of Hepes, 10 mmol L^–1^ of NaOH, 140 mmol L^–1^ of NaCl, and 2.5 mmol L^–1^ of CaCl_2_ at pH 7.4.

### Peptide Stock Solutions

MPX and MP1 were supplied by Aminotech (Diadema, São Paulo, Brazil). The peptides were ordered at a high level of purity (>95%). The homogeneity and sequence were assessed by analytical high-performance liquid chromatography (HPLC) and mass spectrometry (ESI-MS). The peptide stock solutions (2 mmol L^–1^) were prepared in ultrapure distilled water and kept at −20 °C until use.

### Cell Culture

Melan-a (Sigma-Aldrich SCC202) and B16–F10 (ATCC CRL-6475) were kindly donated by M. Galvonas Jasiulionis (Unifesp, Brazil) and M. Jane Zveiter de Moraes (Unifesp, Brazil), respectively. The cells were maintained in RPMI-1640 medium containing 10% FBS, 100 Units mL^–1^ penicillin, and 100 mg mL^–1^ streptomycin in a humidified incubator with 5% CO_2_ at 37 °C. Melan-a was also supplemented with 0.2 μmol L^–1^ of PMA (phorbol-12-myristate-13-acetate) to avoid cell senescence.^28^ Cells were counted using a Neubauer chamber (Neubauer Improved, Olen, Rio de Janeiro, RJ, Brazil) as previously described.^29^ Except for the MTT assay, viable cells were seeded into 24-well plates at a density of 10 × 10^3^ cells/well.

### Cell Viability Assay

Cell viability was assessed using the MTT assay.^30^ Melan-a and B16–F10 cells were separately seeded into 96-well plates at a density of 5 × 10^3^ cells/well. After 24 h, the culture medium was replaced with a serum/antibiotic-free medium. Following, peptides were individually added to each well and maintained for 2 h in a 37 °C humidified incubator with 5% CO_2_. Peptide concentration varied between 5 and 40 μmol L^–1^. After the incubation period, cells were washed, and the medium was replaced with an MTT solution at a final concentration of 50 μg mL^–1^. After 2 h, the solution of each well was replaced by 100 μL of DMSO, and the absorbance of each well was measured at the wavelength of 570 nm on a microplate reader (BioTek Instruments, VT, USA). The cell viability was expressed relative to the mean absorbance of vehicle-treated cells. The half-maximal response concentrations (EC_50_) were determined by fitting data in a dose–response function with a variable slope using the nonlinear regression analysis of GraphPad Prism 8 (GraphPad Software, CA, USA).

### Morphometric Analysis

Changes in cell area and morphology were evaluated by phase contrast microscopy images acquired in an inverted microscope (Nikon Eclipse TiS) equipped with a 40× objective lens. Images were analyzed by manually defining the cell perimeter and measuring it with the ImageJ software. Relative cell area was calculated by dividing each cell area by the mean area of vehicle-treated cells.

### Fluorescence Microscopy Assays

All images were acquired in an inverted fluorescence microscope (Nikon Eclipse TiS) equipped with an LED light source (CoolLed pE-800), a 40× objective lens, and a quad-band filter cube (Chroma 89402 ET). Except the for Annexin V/PI assay, in all other visual experiments, cells were stained according to the respective fluorophore (Table 2), washed with a Hepes-buffered solution (in mmol L^–1^: 20 HEPES, 118 NaCl, 5 KCl, 1.1 MgSO_4_, 1.1 KH_2_PO_4_, 2.5 CaCl_2_, 5.1 α-d-glucose, pH 7.4), and then incubated for an hour with the peptides MPX and MP1 at a final concentration of 20 and 15 μmol L^–1^, respectively. In the case of Annexin V/PI assays, cells were first incubated with the peptides in a binding buffer solution (in mmol L^–1^: 10 HEPES, 10 NaOH, 140 NaCl, 2.5 CaCl_2_, pH 7.4) and then, in the last 15 min of treatment, Annexin V-FITC conjugate (AV-FITC) was added. With no further washing to avoid discarding loose cells, images were immediately captured. PI was added after AV-FITC images were registered.

**Table 2:** Fluorophores Utilized in the Fluorescence Microscopy Assays and Their Attributes^a^

**fluorophore**	**specificity**	**concentration** **(μmol L** ^**–1**^ **)**	**incubation time** (min)	Exc./Em. (nm)
AV-FITC	phosphatidylserine	not specified	15	470/516
DHE	reactive oxygen species	10	30	470/582
Fluo-4	intracellular calcium	2	60	470/515
Hoechst 33342	nuclear DNA (all cells)	1	30	400/455
PI	nuclear DNA (membrane-damaged cells)	10	immediate	550/618
TMRE	mitochondrial membrane potential	0.2	30	550/577

a AV-FITC = Annexin V-FITC conjugate; DHE = dihydroethidium; PI = propidium iodide; TMRE = tetramethylrhodamine ethyl ester perchlorate; Exc./Em. = excitation and emission wavelengths. The concentration of AV-FITC was not specified by the manufacturer, but the fluorophore solution was used in a ratio of 1:100.

For all assays, at least five different fields in each well were analyzed. Each cell or nucleus was selected as a region of interest (ROI) in ImageJ software following these steps: image duplication > threshold (Otsu) > binary > watershed. When necessary, ROIs’ outlines were manually corrected based on the paired phase contrast image. The selection was then transferred to the original image, and the signal was measured in the analyze menu. Fluorescence intensity was calculated offline by subtracting the background mean gray value from the ROI mean gray value. All relative values correspond to data normalized for the vehicle-treated group. The fluorescence index was calculated as the product of the mean fluorescence intensity and the proportion of cells stained with AV-FITC relative to the total cell population. The PI ratio corresponds to the fraction of cells stained with PI relative to the total cell population.

### Statistical Analysis

All assays were performed in at least triplicate. All statistical analyses were conducted using GraphPad Prism 8 (GraphPad Software, Inc.). Significance levels were set at 5%. Data are presented as mean ± SEM (standard error of the mean). Two-group comparisons were performed with the Student’s *t-*test.

## Results

### MPX and MP1 Are Cell-Selective

We first performed MTT assays to analyze and compare the effects of the peptides MPX and MP1 on the cell viability of Melan-a (melanocyte) and B16–F10 (melanoma) cell lines treated with a variety of concentrations for 2 h. MPX and MP1 reduced the viability of both cell lines but more pronouncedly of melanoma cells. As shown in Figure 1A,B, there is an inverse relationship between the peptides’ concentration and cell viability. Moreover, there was a significant difference between the peptides’ half-maximal response concentration (EC_50_) in each cell line. Figure 1C shows that melanocyte cells reached the highest EC_50_ values, indicating that they require higher peptide concentrations to achieve the same viability decrease as in melanoma cells. This can be related to peptides’ physicochemical properties, as well as to cells’ plasma membrane composition. Concentrations near the peptides’ EC_50_ values on melanoma cells were used for all further investigation.

**Figure 1:**
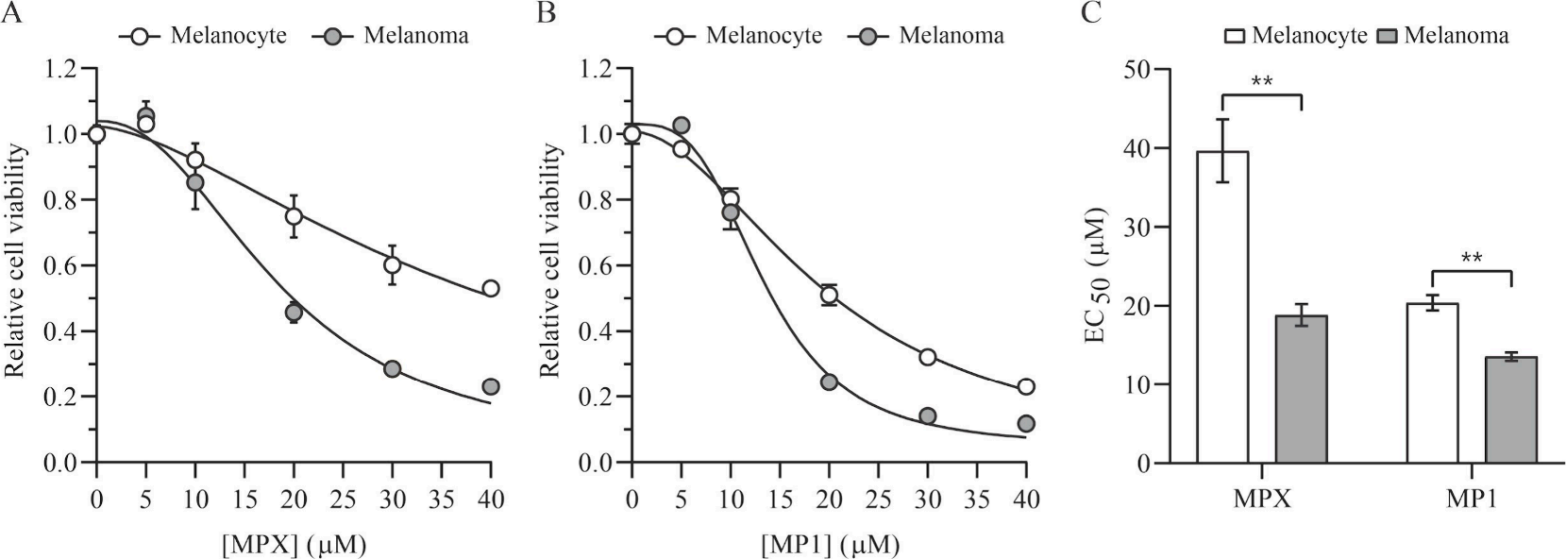
Cell viability of melanocyte and melanoma cells treated with peptides MPX and MP1. The dose–response curve of melanocyte (open circles) and melanoma (filled circles) cells after 2 h of incubation with MPX (A) or MP1 (B) in concentrations ranging from 5 to 40 μmol L^–1^. Data are presented as mean ± SEM of three independent experiments performed in triplicate. (C) Half-maximal response concentration (EC_50_) of peptides MPX (*n* = 3) and MP1 (*n* = 3) in melanocyte (open bars) and melanoma (solid bars) cells. Data are presented as mean ± SEM. Statistical significance was determined by Student’s *t-*tests; **p ≤ 0.01.

### MPX and MP1 Induce Cell Shrinkage and Nuclei Condensation

Following the evaluation of the peptides’ cytotoxicity, we proceeded with morphometric analysis, as morphology observation has traditionally been the primary basis for cell death classification.^31^ Phase contrast image analysis showed that there was a significant morphological and size alteration between cell lines treated for an hour with 20 μM MPX and 15 μM MP1. The images represented in Figure 2A and the analysis in Figure 2B show that compared to vehicle-treated cells, the peptide treatments elicited a cell shrinkage. The area reduction of cells treated with MPX, however, was more intense in melanoma cells. MP1, on the other hand, promoted similar results in both cell lines.

**Figure 2:**
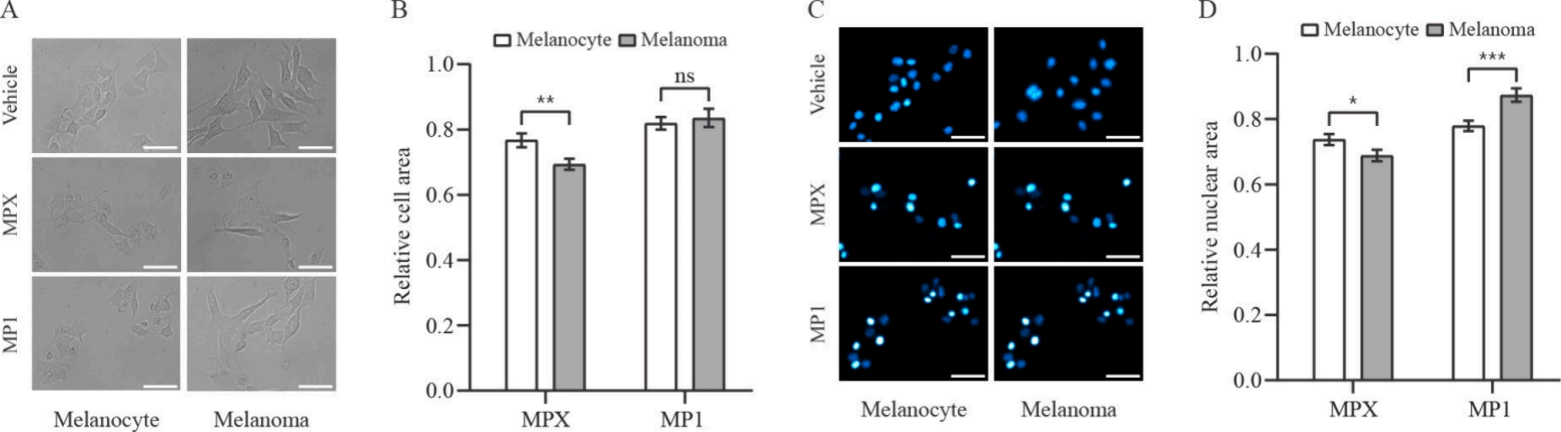
Cell and nuclear morphology of melanocyte and melanoma cells treated with peptides MPX and MP1. (A) Representative phase-contrast microscopy images of melanocyte and melanoma cells incubated for 1 h with vehicle (H_2_O), 20 μmol L^–1^ MPX, and 15 μmol L^–1^ MP1. Scale bar = 50 μm. (B) Quantitative comparison of cell area of peptide-treated melanocyte (open bars) and melanoma (solid bars) cells relative to vehicle-treated cells. Data are presented as mean ± SEM of at least three independent experiments with 5 fields (minimum of 10 cells/field) analyzed for each condition. Statistical significance was determined by Student’s *t-*tests; **p ≤ 0.01; ns, not significant. (C) Representative fluorescence microscopy images of melanocyte and melanoma cell nuclei, (stained with Hoechst 33342, and incubated for 1 h with vehicle (H_2_O), 20 μmol L^–1^ MPX, and 15 μmol L^–1^ MP1. Scale bar = 50 μm. (D) Quantitative comparison of nuclear area of peptide-treated melanocyte (open bars) and melanoma (solid bars) cells relative to vehicle-treated cells. Data are presented as mean ± SEM of, at least, three independent experiments with 5 fields (minimum of 10 cells/field) analyzed for each condition. Statistical significance was determined by Student’s *t-*tests; *p ≤ 0.05; ***p ≤ 0.001.

In addition, fluorescence microscopy was employed to quantify the nuclear area. For that, cells were stained with Hoechst 33342, a membrane-permeant fluorescent dye that stains nuclear DNA of live cells, as shown in Figure 2C. Similar to cell area, nuclear area has decreased in both cell lines after peptide treatments, indicating a nuclear condensation. However, as shown in Figure 2D, MPX and MP1 induced a higher nucleus reduction in melanoma and melanocyte cells.

### MPX and MP1 Do Not Share the Same Antimelanoma Mechanism of Action

Although morphological changes can be shared by more than one type of cell death, the cell membrane state is more distinguishable among them. Thus, plasma membrane organization and integrity were investigated by fluorescence microscopy assays employing Annexin V-FITC and PI staining.

Annexin V is a ligand with high affinity to phosphatidylserine (PS), an anionic phospholipid that becomes externalized on the outer leaflet of plasma membranes as an important physiological signal.^32,33^ We have analyzed the PS exposure of melanocyte and melanoma cells treated for 15 and 60 min with 20 μM MPX and 15 μM MP1 by staining them with Annexin V conjugated with FITC (AV-FITC). As can be seen in Figure 3A–C, peptides MPX and MP1 induced a positive signal for AV-FITC in both cell lines. This signal has increased between 15 and 60 min under both treatments. However, MPX exhibited a more intense signal than did MP1. By comparing melanocyte and melanoma cells, albeit the difference between them after 15 min of treatment with MP1, after 60 min, neither MP1 nor MPX produced a different response between the cell lines. Despite being widely associated with apoptosis, PS exposure is not an exclusive characteristic of this cell death process. Additionally, only necrosis is associated with a fast membrane disruption besides reorganization.^34^ Hence, this assay was followed by a dye exclusion test to assess the membrane integrity.

**Figure 3:**
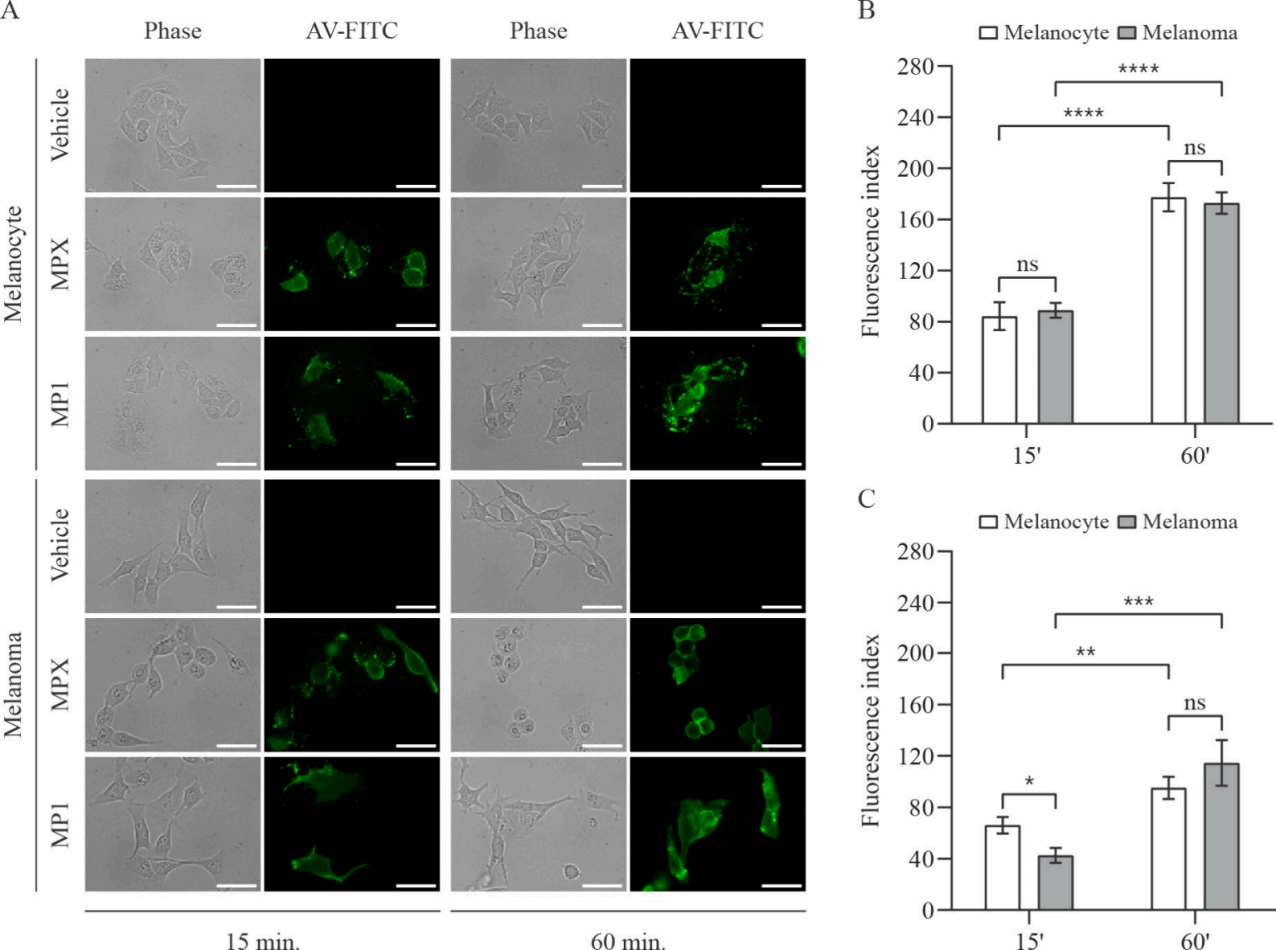
Annexin V detection in melanocyte and melanoma cells treated with peptides MPX and MP1. (A) Representative phase-contrast and fluorescence microscopy images of melanocyte and melanoma cells (stained with Annexin V-FITC conjugate: AV-FITC) incubated for 15 or 60 min with vehicle (H_2_O), 20 μmol L^–1^ MPX, and 15 μmol L^–1^ MP1. Scale bar = 50 μm. Quantitative comparison of the fluorescence index of MPX-treated (B) or MP1-treated (C) melanocyte (open bars) and melanoma (solid bars) cells in 15 and 60 min. Data are presented as mean ± SEM of, at least, three independent experiments with 5 fields (minimum of 10 cells/field) analyzed for each condition. Statistical significance was determined by Student’s *t-*tests; *p ≤ 0.05; **p ≤ 0.01; ***p ≤ 0.001; ****p ≤ 0.0001; ns, not significant. Fluorescence index stands for the product between the mean fluorescence intensity and the ratio of cells stained with AV-FITC relative to the total number of cells.

Propidium iodide (PI) is a cell fluorescent dye and membrane integrity indicator due to its ability to permeate only damaged cells with disrupted cell membranes. Thus, we analyzed the PI uptake of melanocyte and melanoma cells treated with 20 μM MPX and 15 μM for 15 and 60 min. As shown in Figure 4A and B, in 15 min, the PI ratio of melanoma cells treated with MPX was roughly 4-fold higher than that of melanocyte cells. In 60 min, the ratio of PI in melanocyte cells has increased, but not as much as in the melanoma cells. The difference in PI uptake suggests that MPX led to more damage on melanoma than on melanocyte cell membranes. MP1 treatment, however, induced a low ratio of PI on both cell lines, either in 15 or 60 min, as presented in Figure 4A and C. This indicates that MP1 was not as harmful to cell membranes as MPX.

**Figure 4:**
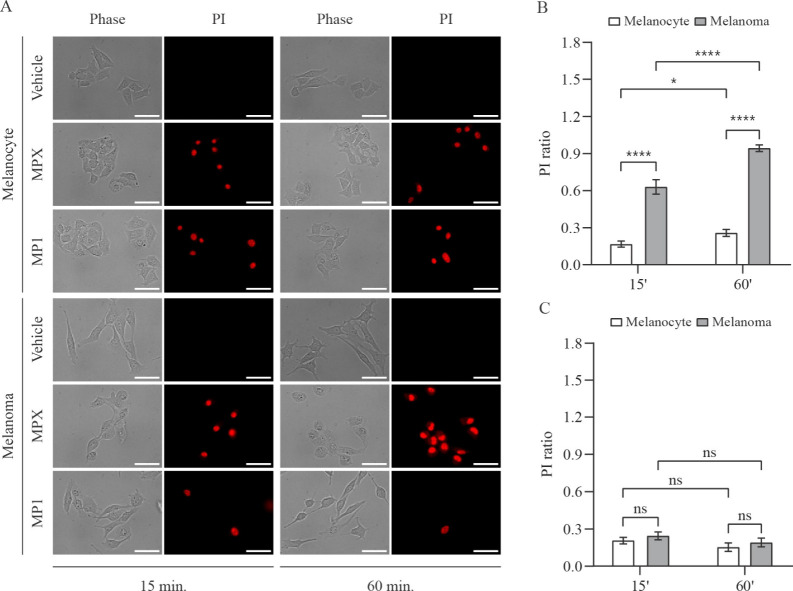
Propidium iodide (PI) uptake of melanocyte and melanoma cells treated with peptides MPX and MP1. (A) Representative phase-contrast and fluorescence microscopy images of melanocyte and melanoma cells (stained with PI) incubated for 15 or 60 min with vehicle (H_2_O), 20 μmol L^–1^ MPX, and 15 μmol L^–1^ MP1. Scale bar = 50 μm. Quantitative comparison of the ratio between PI-stained cells and the total number of MPX-treated (B) or MP1-treated (C) melanocyte (open bars) and melanoma (solid bars) cells in 15 and 60 min. Data are presented as mean ± SEM of, at least, three independent experiments with 5 fields (minimum of 10 cells/field) analyzed for each condition. Statistical significance was determined by Student’s *t-*tests; *p ≤ 0.05; ****p ≤ 0.0001.

Altogether, our results showed that in melanocyte cells, both peptides evoked a PS externalization combined with low PI uptake. In melanoma cells, MP1 promoted this same pattern, but MPX induced PS externalization combined with intense PI uptake. These results combined suggest that the main mechanisms of action triggered by MPX and MP1 peptides are necrosis and apoptosis.

### MPX and MP1 Impair Some Metabolic Pathways

Cell death induces membrane changes but also biochemical imbalance.^35^ Therefore, we have additionally monitored the effect of 1 h MPX and MP1 peptide treatments on reactive oxygen species (ROS), intracellular calcium levels, and mitochondrial membrane potential of melanocyte and melanoma cells. ROS are mediators and regulators of cell metabolism,^36^ calcium ions (Ca^2+^) are signaling agents,^37^ and mitochondrial membrane potential (MMP) is a key factor of cell physiology because cell energy production and, consequently, cell survival depend on it.^38^ These biochemical features were assessed by the mean fluorescence intensities of DHE, Fluo-4, and TMRE.

DHE is a cell-permeant dye that emits fluorescence by binding to ROS, particularly by cytosolic species such as superoxide. Figure 5A and B indicate that there was no significant difference between ROS levels of peptide- and vehicle-treated cells, except for a low decrease in ROS levels of melanocyte cells treated with MPX. Furthermore, only MPX treatment promoted a different response in melanocyte and melanoma cells.

**Figure 5:**
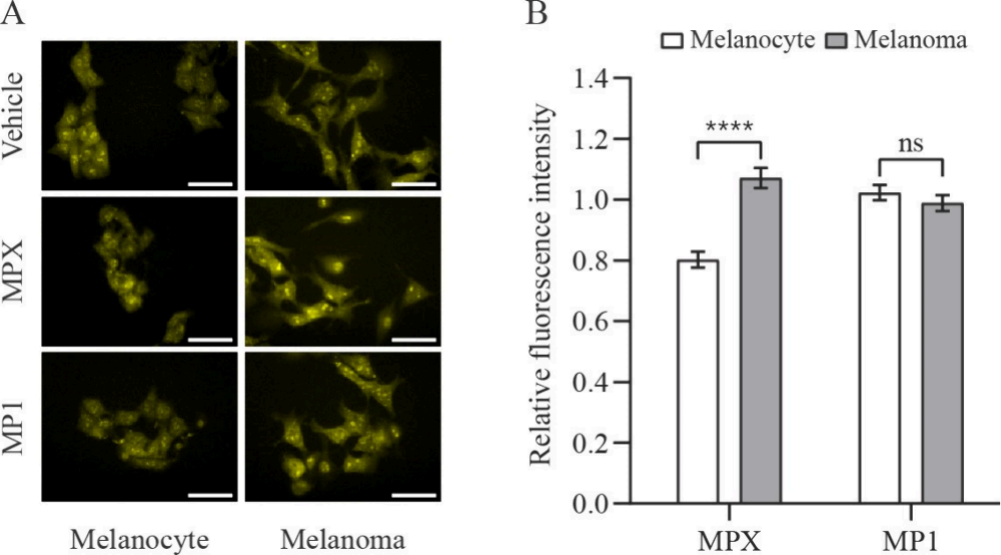
ROS levels in melanocyte and melanoma cells treated with peptides MPX and MP1. (A) Representative phase-contrast and fluorescence microscopy images of melanocyte and melanoma cells (stained with DHE) incubated for 1 h with vehicle (H_2_O), 20 μmol L^–1^ MPX, and 15 μmol L^–1^ MP1. Scale bar = 50 μm. (B) Quantitative comparison of the DHE mean fluorescence intensity of peptide-treated melanocyte (open bars) and melanoma (solid bars) cells relative to vehicle-treated cells. Data are presented as mean ± SEM of, at least, three independent experiments with 5 fields (minimum of 10 cells/field) analyzed for each condition. Statistical significance was determined by Student’s *t-*tests; ****p ≤ 0.0001; ns, not significant.

Fluo-4 is a cell-permeant dye that emits fluorescence by binding to free cytoplasmic Ca^2+^, as illustrated in Figure 6A. It can be seen in Figure 6B that both peptides led to a decrease in intracellular calcium levels of melanocyte cells. In melanoma cells, on the other hand, MP1 treatment did not lead to any significant change, while MPX stimulated an increase in intracellular calcium levels.

**Figure 6:**
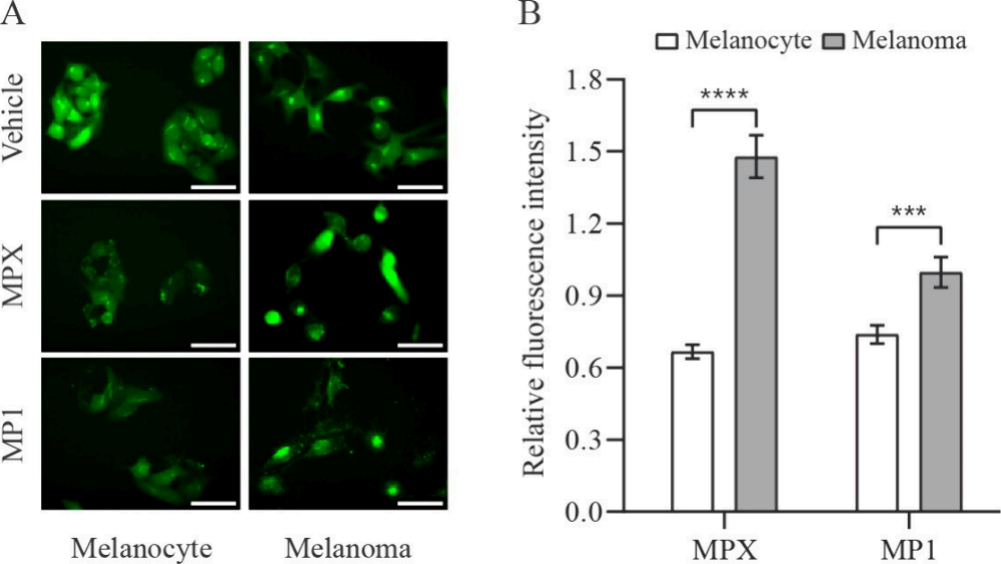
Intracellular calcium levels in melanocyte and melanoma cells treated with peptides MPX and MP1. (A) Representative phase-contrast and fluorescence microscopy images of melanocyte and melanoma cells (stained with Fluo-4) incubated for 1 h with vehicle (H_2_O), 20 μmol L^–1^ MPX, and 15 μmol L^–1^ MP1. Scale bar = 50 μm. (B) Quantitative comparison of the Fluo-4 mean fluorescence intensity of peptide-treated melanocyte (open bars) and melanoma (solid bars) cells relative to vehicle-treated cells. Data are presented as mean ± SEM of, at least, three independent experiments with 5 fields (minimum of 10 cells/field) analyzed for each condition. Statistical significance was determined by Student’s *t-*tests; ***p ≤ 0.001; ****p ≤ 0.0001.

TMRE is a cell-permeable cationic dye that reflects MMP. TMRE has a slow response but is rapidly sequestered by active mitochondria. Increase and decrease in TMRE mean fluorescence indicate, respectively, hyperpolarization (MMP increase) and depolarization (MMP decrease) of mitochondrial membrane.^39^ As illustrated in Figure 7A and B, both cell lines have their MMP decreased after peptide treatments, suggesting that their mitochondria’s membranes had suffered depolarization or total membrane potential collapse. For both peptides, however, this reduction was more intense in melanoma than in melanocyte cells. In addition, MPX elicited a stronger effect than MP1.

**Figure 7:**
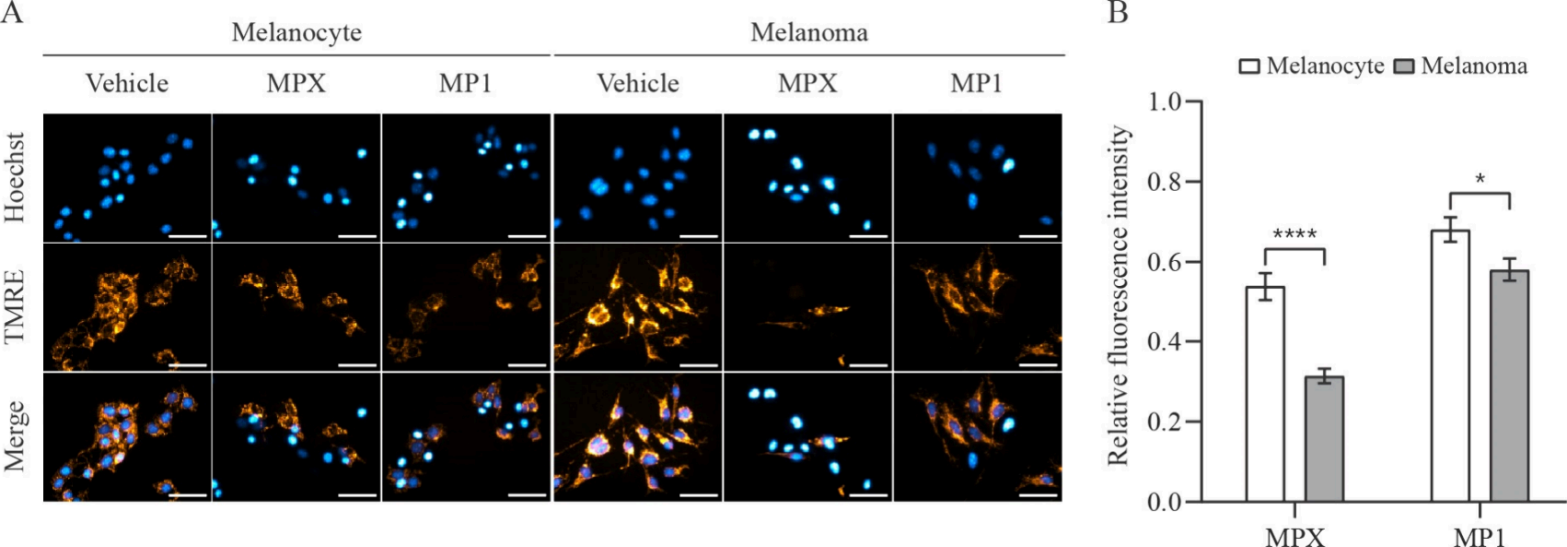
Mitochondrial membrane potential in melanocyte and melanoma cells treated with peptides MPX and MP1. (A) Representative phase-contrast and fluorescence microscopy images of melanocyte and melanoma cells (stained with TMRE) incubated for 1 h with vehicle (H_2_O), 20 μmol L^–1^ MPX, and 15 μmol L^–1^ MP1. Scale bar = 50 μm. (B) Quantitative comparison of the TMRE mean fluorescence intensity of peptide-treated melanocyte (open bars) and melanoma (solid bars) cells relative to vehicle-treated cells. Data are presented as mean ± SEM of, at least, three independent experiments with 5 fields (minimum of 10 cells/field) analyzed for each condition. Statistical significance was determined by Student’s *t-*tests; *p ≤ 0.05; ****p ≤ 0.0001.

These results illustrate the biochemical impairment caused by the peptide treatments and establish intracellular calcium levels and mitochondrial membrane potential as substantial elements of cell selectivity.

## Discussion

Prior studies have indicated that the response to MPX and MP1 peptide treatments might vary among cells.^16,40,41^ This variability may arise from intrinsic differences between tumorigenic and nontumorigenic cells, such as discrepant plasma membrane composition and cell metabolism. The present study has demonstrated that the targeted cell plays an important role in defining the peptide’s mechanism of action. By employing UV–vis spectroscopy and fluorescence microscopy assays, we have elucidated the cytotoxic effects of MPX and MP1 peptides on melanocyte and melanoma cells, revealing that both peptides are cell-selective but elicit different modes of action. These results reassure their anticancer therapeutic potential, highlighting their antimelanoma activity.

Melanoma is the most lethal type of skin cancer due to the high metastatic potential of melanoma cells.^42^ Advanced-stage melanomas are nowadays treated with BRAF/MEK inhibitors or immunotherapy.^27^ However, these treatments often lead to side effects and drug resistance.^26^ The potential cell selectivity and membranolytic action of cationic peptides, well described in biomimetic membranes, make them good candidates in overcoming these issues.^43,44^ In in vitro approaches, MPX and MP1 peptides have already been proven to act against glioblastoma multiforme cells.^16^ Additionally, several reports have shown that MP1 acts against leukemic T-lymphocyte cells and prostate, bladder, and lung cancer cells.^18,19,40^ Our findings indicate that MPX and MP1 peptides are also cytotoxic to melanoma cells, and remarkably, this cytotoxicity is more intense in melanoma than in melanocyte cells, indicating that they are cell-selective. To our knowledge, this is the first report about MPX and MP1 peptide cell selectivity in syngeneic adherent cells, i.e., derived from genetically identical cell types.

The current investigation found that almost all analyzed parameters were divergent between melanocyte and melanoma cells, indicating that the peptides’ selectivity may result from the combination of all physical and chemical modifications. Although these findings were not enough to determine a single responsible factor for cell selectivity, they shed light on the mechanism of action of each peptide. Previous studies evaluating their cytotoxicity mechanism indicate that, mostly, their mode of action is related to necrosis.^16,18^ However, it has also been found that MP1 has stimulated apoptosis in A549 cells.^19^

For a long time, the primary basis for cell death classification has relied on the recognition of cell morphology alterations.^35^ Our findings show that MPX and MP1 peptides led to a decrease in cell and nuclear areas. Although cell shrinkage and nuclei condensation are classic hallmarks of apoptosis,^45^ it should be noted that different types of cellular insults can produce similar morphological features. For instance, pyknosis, an irreversible condensation of the nucleus, has been associated with both apoptotic and necrotic cell deaths.^46^ Therefore, the morphological data presented in this study do not provide mechanistic insights on their own. They do allow, however, the visualization of the differential peptides’ effect on both cells, which was only numerically demonstrated in the previous cell viability assays.

Beyond morphological analysis, cell death classification has long been resolved by analyzing the cell plasma membrane state.^47^ Annexin V-FITC conjugate is a phospholipid-binding fluorescent protein with a high affinity for phosphatidylserine (PS), a phospholipid that is translocated to the external surface of the plasma membrane during necrosis and apoptosis. Propidium iodide (PI), on the other hand, is an exclusion dye of cell membrane integrity.^32^ In this study, we used these fluorophores in microscopy assays in order to visualize the membrane damage caused by the peptides. Our findings show that MPX and MP1 peptide treatments resulted in PS exposure but only MPX led to significant combined PI uptake. This may indicate that only MPX-treated melanoma cells suffered from a membranolytic action.

It has already been shown that some cell membrane and organelle damage is related to increased levels of ROS due to the action of external or internal agents.^48^ Although our results indicate a plasma membrane disturbance, we have seen no alteration in ROS levels in cells treated with either MPX or the MP1 peptide, except for a little decrease in MPX-treated melanocyte cells. We hypothesize that this result does not determine an absent involvement of ROS in the metabolic pathways impaired by these peptides, but rather that the specific ROS assessed by the fluorophore DHE were not altered. Cell damage has also been related to altered levels of intracellular calcium and to mitochondrial dysfunction.^49–51^ In accordance with the peptides gomesin, melittin, and temporin-1CEa that are, respectively, cytotoxic to CHO,^52^ A549,^53^ and MDA-MB-231 cells,^54^ MPX induced an increase in intracellular calcium levels combined with a decrease in mitochondrial membrane potential of melanoma cells. In melanocyte cells, MPX led to a decrease in intracellular calcium levels and also a decrease in the mitochondrial membrane potential. This decrease, however, was not as intense as that in melanoma cells. This latter behavior was also noticed in MP1-treated melanocyte and melanoma cells. It is possible that the mitochondrial membrane depolarization elicited by the peptides is the result of pores formed on either the mitochondria’s inner or outer membrane.^51,55,56^

Although cell death mechanisms share some of the same cell modifications, differing only on how they reach them, collectively, our results indicate that MPX and MP1 peptides drive necrosis and apoptosis in melanoma cells and apoptosis in melanocyte cells. Figure 8 schematizes the main events related to these processes.

**Figure 8:**
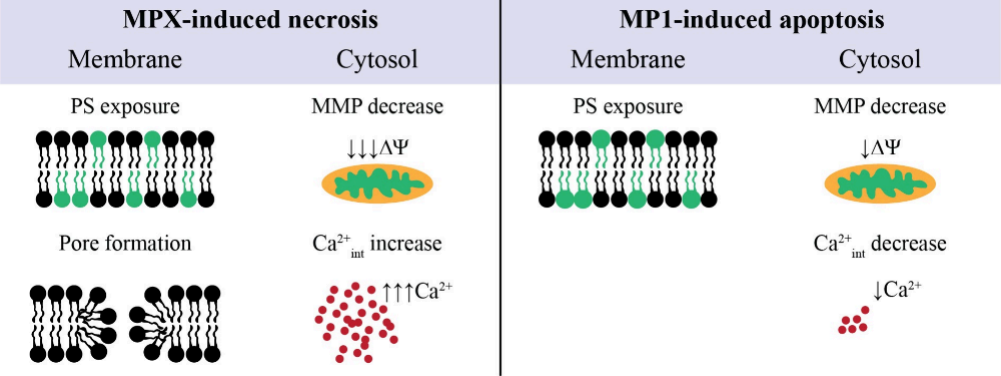
Schematic mechanistic interpretation. Main cell membrane and cytosolic events related to necrosis and apoptosis induced by MPX and MP1 peptides in melanoma cells.

The type of cell death induced by the peptides might be a trade-off between their physicochemical properties and their cell attributes. Previous works with biomimetic membranes showed that α-helical amphiphilic peptides are capable of translocating across phospholipid bilayers, of forming transmembrane pores, or even of acting synergistically.^57^ This observation aligns with reported CD spectra indicating that MPX and MP1 peptides adopt helical structures in neutrally and negatively charged lipid membranes.^16^ Moreover, it has widely been shown that these peptides exhibit a higher affinity to anionic liposomes composed of PCPG (phosphatidylcholine with phosphatidylglycerol) or PCCL (phosphatidylcholine with cardiolipin) than to the zwitterionic ones only composed of PC (pure phosphatidylcholine) or PCC (phosphatidylcholine with cholesterol).^58–60^ The same was observed for PCPS vesicles (phosphatidylcholine with phosphatidylserine) that mimic cancer cell membranes. Thus, these membrane-binding studies may indicate how the peptide selectivity, demonstrated here in cells, arises. Additionally, there is evidence that MPX and MP1 peptides increase the bilayer permeability of anionic membranes, originating from lipid-peptide clusters. These clusters, however, are more easily observed in liposomes treated with MPX rather than with MP1.^60^ This may explain why each peptide led to a different cell death mechanism in melanoma cells.

Increased hydrophobicity of peptides has also been related to enhanced cytotoxicity of cancer cells due to their consequently improved membrane disruption capacity.^61–63^ Once the MPX peptide is more hydrophobic than the MP1 peptide, it is possible that the former triggers necrosis, because it stays confined to the cell membrane, and that the latter triggers apoptosis by being able to reach intracellular targets by penetrating the cell. In accordance with this hypothesis, a previous study has proven that the higher the peptide net charges, the stronger the membrane affinity.^64^ Thus, the higher net charge of MPX compared to that of the MP1 peptide might be another reason for its membranolytic activity. Nonetheless, MP1 has previously been reported to disrupt K562 and K562/ADM cell membranes.^41^ These contradictory findings might be a consequence of the cell specificities. Although anionic molecules are found in most cells, cancer cells express higher levels of some glycoproteins, glycolipids, and proteoglycans.^17^ Additionally, these cells often expose anionic lipids, such as phosphatidylserine (PS), to their outer membrane leaflet.^65^ These features change the peptide-cell interaction and, hence, can contribute to the divergent observations regarding peptides’ mode of action.

In the context of medical purposes, the peptides’ cell selectivity underscores their potential therapeutic use against melanoma cells. Nonetheless, some challenges still remain for clinical translation. MPX, for example, can be conveniently administered by the oral route^66^ but cannot be delivered in high concentrations due to its hemolytic activity.^10^ MP1, on the other hand, is less toxic for erythrocytes, exhibiting almost no hemolytic activity,^11^ but it is highly susceptible to proteolysis.^67^ Despite these issues, a number of approaches are already available for addressing them, such as selective acylation,^68^ hydrophobicity modulation,^69^ and structural stability modification.^67^ Moreover, the peptides MPX and MP1 are still good candidates to be included in the pharmaceutical landscape because of their comparatively low effective concentrations. A variety of other peptides have been investigated for their antimelanoma activity. Nisin Z^70^ and Chartergellus-CP1,^71^ for example, were both selective to melanoma cells. However, the concentration required for this cell selection was considerably higher than the concentration required for mastoparan peptides. Additionally, some cell-type intrinsic aspects might be set aside in those investigations because of the nonsyngeneic cell models employed, i.e., HaCaT (nonmalignant keratinocytes) and A375 (melanoma) cell lines.

In view of this, beyond advancing the understanding of the anticancer action of MPX and MP1 peptides, this study highlights their property of cell selectivity in a syngeneic cell model, reassuring their antimelanoma therapeutic potential and accounting for cell-type-specific variability instead of comparing the cancerous cell to a generic normal cell. Moreover, our findings suggest that the peptides’ mode of action depends not only on their physicochemical properties but also on their target characteristics. We conclude that both of these variables must be considered in future investigations.
